# A Wireless Sensor Network for High Spatial and Temporal Resolution Soil Gas Emission Monitoring

**DOI:** 10.3390/s26144605

**Published:** 2026-07-20

**Authors:** Yoganand Biradavolu, Hendri Yuda Winanto, Muhammad Osama Shahid, Bhuvana Krishnaswamy, Jingyi Huang

**Affiliations:** 1 Department of Electrical and Computer Engineering, University of Wisconsin–Madison, Madison, WI 53706, USA; bhuvana@ece.wisc.edu; 2 Department of Soil and Environmental Sciences, University of Wisconsin–Madison, Madison, WI 53706, USA; hendri.yuda@pupuk-indonesia.com (H.Y.W.); jhuang426@wisc.edu (J.H.); 3 PT Pupuk Indonesia (Persero), Jakarta 11480, Indonesia; 4 Department of Computer Science, Wayne State University, Detroit, MI 48202, USA; mshahid2@wayne.edu

**Keywords:** soil CO_2_ monitoring, wireless sensor networks, carbon flux measurement, low-power agricultural sensing, spatio-temporal environmental monitoring

## Abstract

Wide-scale, spatio-temporal quantification of soil CO_2_ efflux is essential for understanding terrestrial carbon dynamics, predicting climate change, and evaluating the carbon balance in managed and natural ecosystems. Rising global temperatures, changing land use patterns, and other activities aimed at boosting crop productivity have resulted in an increase in microbial activity, increasing the impact of soil on gas exchange. Therefore, it is important to measure ${\mathrm{CO}}_{2}$ gas exchange in situ, over wide areas and extended periods without manual intervention. However, current approaches such as remote sensing lacks sufficient spatial and depth resolution, while other direct measurements such as eddy covariance demand expensive infrastructure, limiting wide-scale deployment. In this work, we propose a low-cost, battery-operated ${\mathrm{CO}}_{2}$ sensing system that provides long-term and scalable monitoring of soil respiration and carbon flux, with the promise for high-resolution measurements. Our innovative design features a PVC-based gas chamber that periodically opens and closes to allow for gas exchange, and a sensor module with low-cost temperature, moisture, pressure, and ${\mathrm{CO}}_{2}$ sensors, with a low-power wireless LoRa network for real-time monitoring. Our system was rigorously validated through multiple outdoor deployments, over long periods to demonstrate its practicality. We observe that temperature, air pressure, and humidity trends show responsiveness to the environment. We also observe that ${\mathrm{CO}}_{2}$ emission flux rate vary significantly across different modules, underscoring the need for fine-grained spatial and temporal resolution in monitoring.

## 1. Introduction

Carbon dioxide (${\mathrm{CO}}_{2}$) is one of the most important greenhouse gases on Earth, helping to maintain the global average surface temperature above freezing point by absorbing long-wave radiation. However, the rapid rise in atmospheric ${\mathrm{CO}}_{2}$ concentration—from approximately 300 ppm to over 430 ppm in the past century—has become a major driver of climate change [1,2]. Among natural sources, soil respiration contributes significantly to atmospheric ${\mathrm{CO}}_{2}$, releasing an average of 6 g per square meter per day globally [3]. Within terrestrial ecosystems, both autotrophic respiration (AR) and heterotrophic respiration (HR) are dominant contributors to this efflux [2,4]. Accurate quantification of soil ${\mathrm{CO}}_{2}$ efflux is crucial for understanding terrestrial carbon dynamics, predicting climate change trajectories, and evaluating the carbon balance in managed and natural ecosystems [5].

Soil plays a vital role in the global carbon cycle, serving as a crucial intermediary between the atmosphere and the biosphere. It is estimated that soils store approximately three times more carbon than is found in either the atmosphere or terrestrial vegetation [6]. Microbial activity in soils, particularly HR, has shown an increasing trend over the past two decades, likely due to rising global temperatures and changing land use patterns [7]. However, the dynamics of ${\mathrm{CO}}_{2}$ emissions resulting from AR/HR are complex and require an in-depth study to identify the major driving factors. This complexity arises from the significant spatio-temporal variability in soil ${\mathrm{CO}}_{2}$ flux, which differs across ecosystems such as forests, grasslands, croplands, and pastures [8]. These fluxes can also vary on daily and seasonal timescales, governed by fluctuations in environmental drivers such as temperature, moisture, and photosynthetic activity [6,9,10]. One of the primary drivers of the variations in soil ${\mathrm{CO}}_{2}$ efflux is the strong dependence of heterotrophic respiration (HR) on complex and dynamic interactions between physical and chemical soil properties. Among these, soil temperature and moisture play a dominant role in regulating respiration rates [8]. Additionally, land management practices such as tillage, irrigation, and fertilizer application, commonly adopted to enhance crop yields in agricultural lands, further alter the physical and chemical properties of soil. Barnard et al. [11] emphasize that rewetting dry soils can trigger a pronounced pulse in ${\mathrm{CO}}_{2}$ efflux, a phenomenon known as the Birch Effect, with significant global implications. The combined effect of fertilizer application and soil rewetting often leads to a spike in carbon and nitrogen mineralization, promoting microbial activity and intensifying both HR and AR. This underscores the need for accurate quantification of soil respiration, and hence ${\mathrm{CO}}_{2}$ efflux, across diverse land cover/use types to improve predictions of future atmospheric ${\mathrm{CO}}_{2}$ concentrations.

**Existing Research:** Current techniques for measuring ${\mathrm{CO}}_{2}$ emissions broadly fall into two major categories: (1) remote sensing-based methods, and (2) in situ measurement techniques.

Remote sensing methods include airborne and satellite-based systems [3], which offer wide-area coverage and are particularly useful for tracking emissions in hard-to-reach locations such as volcanic regions. More precise ground-based methods like Differential Absorption LIDAR and Solar Open-Path Fourier Transform Spectroscopy provide better resolution [5], but these systems are expensive—costing thousands of dollars—and lack the sensitivity and fine-grained temporal or spatial resolution needed for many applications.

In situ methods involve direct measurements at or near the emission source. Among them, the eddy covariance method [12,13] estimates flux by computing the covariance between vertical wind fluctuations and variations in gas concentration. While this technique provides useful large-scale measurements, it typically requires tall tower installations, making it expensive, labor-intensive, and limited in spatial resolution (typically a few hundred meters). Scaling it for broader deployment would require significant physical and financial resources. Moreover, this method may overestimate fluxes; for example, Pastorello et al. [14] report that eddy covariance tends to measure fluxes approximately 22.5% higher than chamber-based methods. Chamber-based techniques offer high accuracy but suffer from limited spatial coverage, as they typically measure flux at only a single point [15,16]. While some studies such as Harmon et al. [17] have proposed low-cost ${\mathrm{CO}}_{2}$ monitoring systems, they too are constrained to single-point measurements and do not offer scalable, high-resolution spatial mapping [18,19,20].

Due to these limitations, studies on ${\mathrm{CO}}_{2}$ estimation have been limited in their spatio-temporal measurement coverage. Therefore, there is a clear need for a low -cost and accurate ${\mathrm{CO}}_{2}$ sensing system with a small form-factor that can be deployed at scale in order to measure ${\mathrm{CO}}_{2}$ flux variations by (i) capturing spatial variations in ${\mathrm{CO}}_{2}$ flux across large areas with fine-grained resolution, and (ii) monitoring temporal variations with high resolution over extended periods (on the order of years).

In this work, we present a novel low-cost, battery-operated ${\mathrm{CO}}_{2}$ sensing system that enables long-term and scalable monitoring of soil respiration and atmospheric carbon flux. A low-cost system has the potential to scale and provide high-resolution estimates for a large field. Such a scalable system could be used in conjunction with other commercial systems to provide more ${\mathrm{CO}}_{2}$ data to identify the hot spots and hot moments of ${\mathrm{CO}}_{2}$ fluxes across large spatiotemporal extents, rather than obtaining absolute ${\mathrm{CO}}_{2}$ flux measurements. Our key contributions include the following:

**Key Contributions:**

- We design an ultra-low-power ${\mathrm{CO}}_{2}$ sensing system that could potentially operate for years on off-the-shelf batteries.
- Our architecture supports scalable deployment of tens of modules for fine-grained spatial coverage.
- We integrate additional sensors (soil moisture, air/soil temperature, air pressure) to capture environmental context.
- We design a wireless system that supports long-range communication using the LoRa, enabling sensors to transmit data reliably to base stations.
- Our system enables long-term, real-time ${\mathrm{CO}}_{2}$ monitoring for digital agriculture and climate science.

## 2. Materials and Methods

In this section, we present the design of our end-to-end ${\mathrm{CO}}_{2}$ sensing system. We begin by detailing the sensor module architecture, which integrates multiple environmental sensors in a custom designed PCB, and a chamber for gas-exchange. We then describe the wireless communication pipeline used to transmit data to a remote base station, followed by the power optimization strategies that enable long-term deployment. Finally, we describe the calibration procedure for the ${\mathrm{CO}}_{2}$ sensor.

### 2.1. Sensor Module Design

In this section, we describe the design and integration of our sensor modules for monitoring environmental variables critical to soil ${\mathrm{CO}}_{2}$ analysis. Each module combines multiple low-power sensors with a custom-designed PCB optimized for long-term battery operation. The sensing unit is enclosed in a specially designed gas chamber that permits ambient air exchange while protecting the electronics from environmental exposure. A low-power radio is interfaced with the board to wirelessly transmit data to a centralized base station using LoRa protocol. We detail the module architecture and explain our design choices in the following subsections.

**Figure 1:**
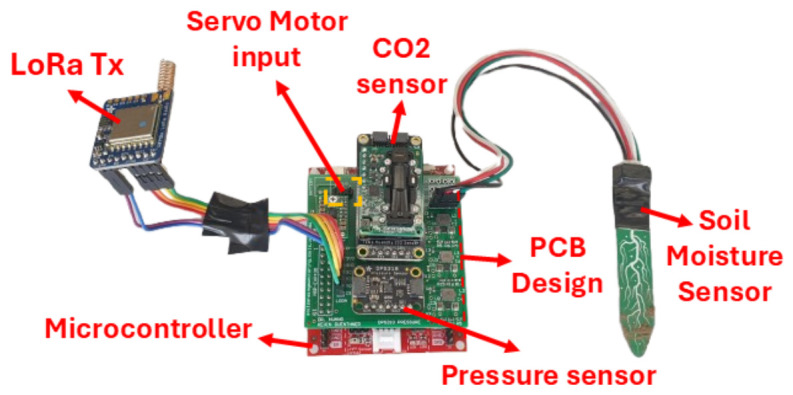
Sensor module with integrated sensors, microcontroller and LoRa radio on a custom designed PCB.

**Sensing Hardware:** We design the sensor module to integrate multiple sensors, each dedicated to measuring a specific environmental variable. These sensors are connected via a custom-designed PCB, as illustrated in Figure 1. As discussed in Section 1, soil moisture, temperature, and humidity play critical roles in estimating ${\mathrm{CO}}_{2}$ flux, as they influence both heterotrophic and autotrophic respiration. Therefore, measuring these variables alongside ${\mathrm{CO}}_{2}$ enables more accurate modeling of soil respiration dynamics. To measure soil moisture, we use the Adafruit STEMMA Soil Sensor [21]. Air pressure is recorded using the DPS310 barometric pressure sensor [22], and carbon dioxide concentration is measured using the Adafruit SCD-30 non-dispersive infrared (NDIR) ${\mathrm{CO}}_{2}$ sensor [23], which also includes integrated temperature and humidity sensors. All sensors are powered via a 3.3 V voltage regulator [24]. The selection of these components was guided by low power consumption and cost-effectiveness to support long-term operation without frequent battery replacements. The SCD-30 ${\mathrm{CO}}_{2}$ sensor has a calibrated measurement range of 400–10,000 ppm and a specified accuracy of ±(30 ppm + 3% of the measured value). The DPS310 operates over a pressure range of 300–1200 hPa with precision up to ±0.002 hPa in high-precision mode, while the soil moisture sensor reports approximate raw values ranging from 200 (very dry) to 2000 (very wet).

**PCB Design:** As shown in Figure 1, we designed a PCB layout that houses connections to all the sensors, the servo motor, and the LoRa radio for wireless data transmission (details in Section 2.4). The ${\mathrm{CO}}_{2}$ and pressure sensors are directly mounted and soldered onto the PCB, while the soil moisture sensor, servo motor, and LoRa radio are connected via jumper wires. This allows the moisture sensor to be buried in the soil and the radio to be oriented optimally for long-range transmission. The PCB also includes integrated 3.3 V and 5 V voltage regulators to supply appropriate voltage levels to each component. The entire PCB stack is interfaced with a TI MSP430FR2355 microcontroller [25], which communicates with the sensors over SPI and I^2^C protocols.

**Figure 2:**
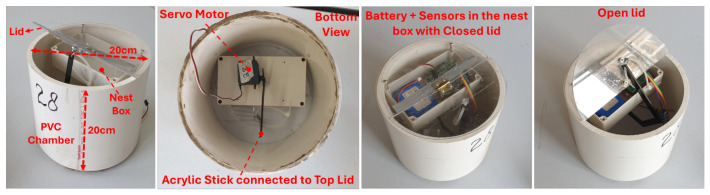
Sensor module design with PVC chamber, servo-controlled lid, and sensors housed inside.

**Structural Design:** To house the sensing electronics and enable controlled gas exchange, we designed a protective gas chamber structure using a PVC cylinder with a diameter and height of 20 cm, as shown in Figure 2. Inside each chamber, we mounted a small nested box to securely hold the sensing PCB, wireless transmitter, and power source. One end of the cylinder is sealed with a transparent polycarbonate lid connected to a servo motor [26] via an acrylic linkage. The servo motor opens or closes the lid to allow regulated air exchange. The opposite end remains open to allow the soil moisture sensor to be inserted into the soil. The enclosure serves a dual purpose: it enables regulated exposure of the sensors to the surrounding environment (e.g., for ${\mathrm{CO}}_{2}$ accumulation and release cycles), and protects the electronic components from environmental stressors such as rain, wind, and physical debris.

### 2.2. Firmware and Control Logic

**Figure 3:**
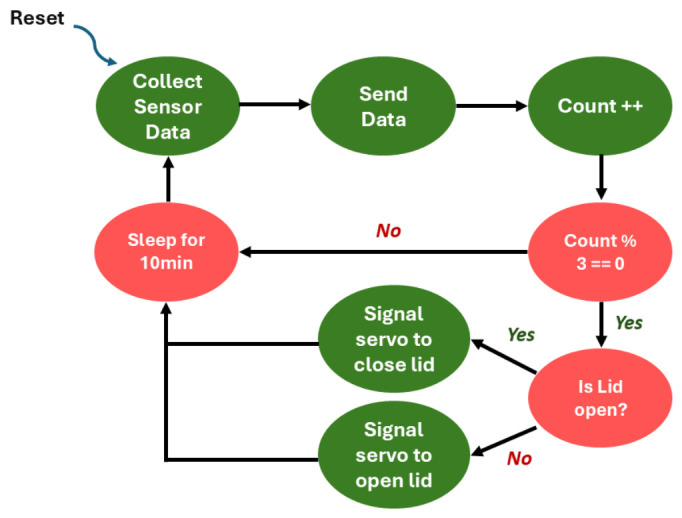
Flow diagram of microcontroller code.

The firmware running on the microcontroller governs all core sensing and actuation tasks, including sensor data acquisition, lid control, power management, and wireless data transmission. We implemented this logic in embedded C++ and flashed it onto a TI MSP430FR2355 microcontroller [25]. Upon powering up (e.g., after connecting the battery), the system enters a reset state and begins its sensing cycle. The microcontroller initiates I^2^C communication with each sensor module and waits 10–15 s for them to stabilize and provide valid readings. After acquiring all sensor values, it formats the data and transmits it via the onboard LoRa module using SPI communication (detailed in Section 2.4). To support long-term operation and periodic gas exchange, the firmware enforces a strict measurement and actuation schedule: the sensor module wakes up every 10 min to sense and transmit data, and alternates lid position every 30 min. This is implemented using a simple counter-based algorithm, illustrated in Figure 3. Each time data is sent, a counter variable is incremented. If the current count modulo 3 is zero (i.e., every third cycle), the system toggles the lid state—opening or closing it via PWM control of the servo motor. Otherwise, the device enters a low-power sleep mode for the next 10 min, where all sensors are powered down and the MCU enters its lowest-power state. A watchdog timer is used to wake the device and begin the next cycle. This design ensures that ${\mathrm{CO}}_{2}$ accumulation and air exchange cycles alternate every 30 min, while minimizing energy consumption between sensing events.

### 2.3. Power Optimization

To maximize battery life, the microcontroller enters a low-power sleep mode between sensing cycles, using a watchdog timer to wake every 10 min. During sleep, power to all sensor modules is cut, and only the internal low-frequency oscillator remains active. Through careful firmware scheduling and regulator selection, we reduced the system’s sleep current to approximately 0.08 mA. When active—i.e., during sensing, data transmission, or servo operation—the average current draw is around 70 mA. Over a 30-min cycle, the effective average current consumption is approximately 1.1 mA. We power the system using a 3.7 V 6600 mAh lithium-ion battery [27]. Based on the measured current profile, the system can operate continuously for nearly two months without requiring a battery replacement.

### 2.4. Wireless Communication Using LoRa

To enable long-range wireless communication between each sensor module and a centralized base station, we use the RFM95 LoRa transceiver [28], which operates in the unlicensed 915 MHz ISM band. LoRa [29] is a widely used low-power wide-area network (LPWAN) protocol capable of supporting communication over several kilometers with data rates of up to a few kilobits per second. This makes it well-suited for our application, where sensor modules are deployed over large areas, transmit small packets infrequently, and require energy-efficient communication. Our deployment uses a single-hop star topology, where all sensor modules communicate directly with a single centralized base station. Each sensor module packages its measurements into LoRa packets along with a unique module ID and transmits them to the base station upon each sensing cycle. For channel access, the sensor modules use an ALOHA-based medium access control with Channel Activity Detection (CAD) for uplink transmissions. Because each module transmits infrequently and the aggregate channel occupancy is low, CAD was enabled to reduce the probability of packet collisions in our deployment. The LoRa physical-layer parameters were selected to prioritize communication range while maintaining low power consumption. In our deployment, we used the 915 MHz ISM frequency band with a spreading factor of SF10, coding rate of 4/5, bandwidth of 250 kHz, and transmit power of 20 dBm, which is the maximum supported by the hardware. These parameters were selected using LoRa range estimation tools [30], and then validated in our field deployment. More details on generalizing these parameters are discussed in Section 4.

**Figure 4:**
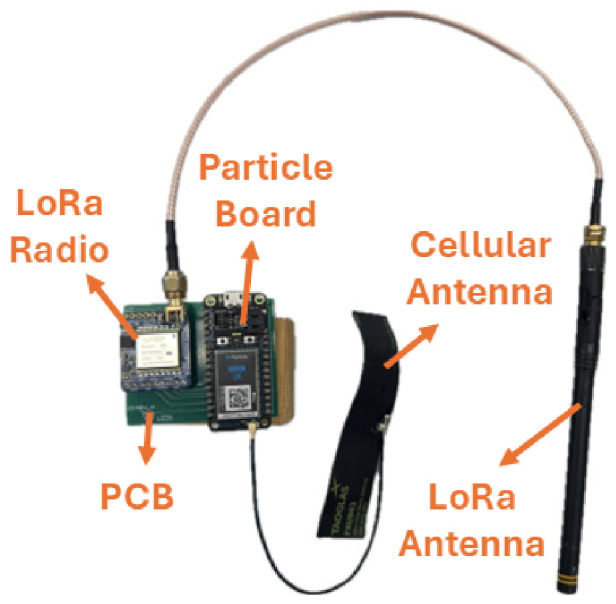
Receiver hardware with Particle Boron and LoRa radio.

The base station, implemented using a Particle Boron Board [31] and shown in Figure 4, receives these packets, decodes them, and forwards the parsed data to the cloud via its integrated LTE connectivity. A spring antenna is used on both ends to enhance communication range, achieving reliable transmission distances of up to 1 km in open terrain. To ensure long-term autonomous operation, the base station is powered by a solar panel, which continuously charges a connected battery pack. This setup allows the receiver to remain operational in remote outdoor locations without manual recharging. Additionally, the base station hardware is housed inside a waterproof and weather-resistant enclosure to protect against rain, dust, and environmental damage during prolonged field deployments.

### 2.5. CO_2_ Sensor Calibration

NDIR-based ${\mathrm{CO}}_{2}$ sensors, such as the Adafruit SCD-30 [23], require calibration against a reference-grade instrument prior to field deployment to ensure accurate measurements. We perform this calibration using a linear regression model trained against a benchmark sensor.

**Calibration Setup.** We place up to nine ${\mathrm{CO}}_{2}$ sensor modules simultaneously inside a sealed calibration gas chamber, where ambient ${\mathrm{CO}}_{2}$ levels are gradually increased using compost over a 12 h period. As a reference, we use the Vaisala CARBOCAP® Hand-held Carbon Dioxide Meter GM70 (Vaisala Oyj, Vantaa, Finland) [32], which provides accurate, real-time ${\mathrm{CO}}_{2}$ concentration readings. Each sensor’s raw readings are then linearly regressed against the benchmark data to obtain a unique calibration model per unit.

**Calibration Model.** The calibration follows the linear model shown in Equation (1), where the benchmark concentration *y* is modeled as a function of the uncalibrated sensor reading $x_{1}$:

(1) $$\begin{array}{ccc} y & = & \beta_{0}+\beta_{1}x_{1}+\epsilon \end{array}$$

Here, $\beta_{0}$ and $\beta_{1}$ are the regression intercept and slope for each sensor, and ϵ denotes the residual error. This regression equation is used to transform future raw ${\mathrm{CO}}_{2}$ sensor outputs into calibrated concentration values in ppm.

**Figure 5:**
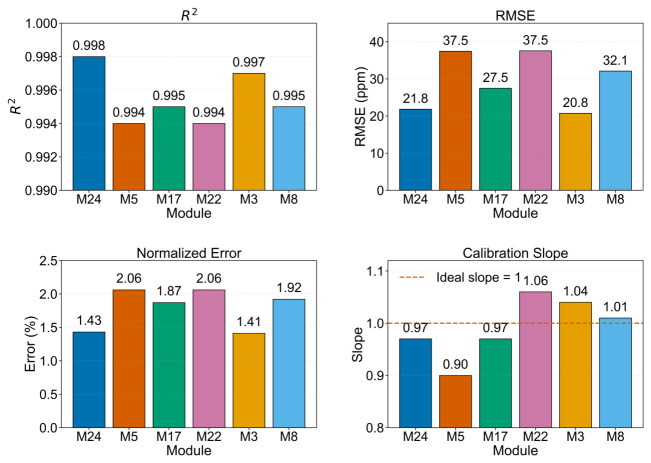
Calibration performance summary for the representative modules used in the field analysis, showing high $R^{2}$, low RMSE, and low normalized error across modules.

Table 1 summarizes the regression coefficients and calibration performance for the representative modules used in the field analysis. The slopes $\beta_{1}$ are close to the ideal value of 1, while the intercepts $\beta_{0}$ vary across modules, indicating the need for module-specific calibration. Across these modules, the mean $R^{2}$ is 0.996, the mean RMSE is 29.53 ppm, and the mean normalized error is 1.79%, showing consistently high calibration accuracy. Figure 5 provides a visual summary of these calibration metrics.

**CO_2_ Flux Estimation.** Once calibrated, the sensor readings can be used to compute the soil ${\mathrm{CO}}_{2}$ flux rate using Equation (2):

(2) $$\begin{array}{ccc} F & = & \frac{PV}{RTA}\times \frac{dC}{dt} \end{array}$$

where

- *F*: ${\mathrm{CO}}_{2}$ flux rate (μmol $m^{-2}\;s^{-1}$);
- *P*: Barometric pressure (kPa);
- *V*: Volume of the flux chamber (L);
- *R*: Universal gas constant (8.314 L kPa $K^{-1}\;{\mathrm{mol}}^{-1}$);
- *T*: Air temperature (K);
- *A*: Soil surface area enclosed by the chamber ($m^{2}$);
- $\frac{dC}{dt}$: Time derivative of ${\mathrm{CO}}_{2}$ concentration (ppm/s).

The flux rate calculation requires time series data from the calibrated ${\mathrm{CO}}_{2}$ sensor, along with synchronized measurements of pressure, temperature, and chamber volume and area. This formulation enables continuous, in situ estimation of soil respiration in terms of ${\mathrm{CO}}_{2}$ efflux.

## 3. Results

**Figure 6:**
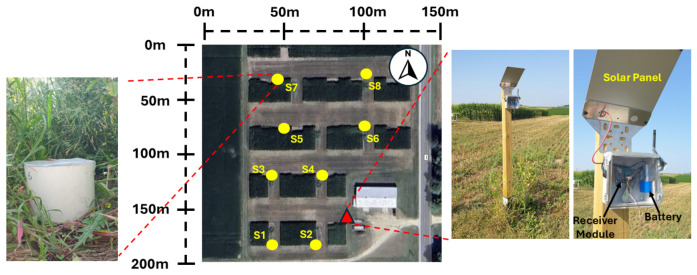
Field deployment layout with 12 sensor modules distributed across eight stations in an agricultural field and a solar-powered receiver mounted on a wooden pole.

We evaluate our system by deploying it on an agricultural field located in Wisconsin, USA, measuring approximately 200 m × 150 m, as shown in Figure 6. The experimental field for the sensor network is generally flat with an elevation ranging from 302 to 308 m. The entire field was planted with the same crop (corn, *Zea mays* L.) and was managed uniformly, with the same planting and harvesting dates, seed density, and fertilizer rates. The study field has a predominantly silt loam soil texture, and differences in soil temperature may be attributable to spatial heterogeneity in soil moisture arising from variations in micro-topography and soil texture.

We selected eight stations 1, 2, …, 8 (denoted by yellow circles) as deployment points. Stations 3 through 6 each hosted one sensor module, while stations 1, 2, 7, and 8 hosted two modules each, resulting in a total of 12 deployed modules. As shown in the figure, the lower ends of each module were buried in the soil to serve as a gas chamber. The receiver (marked by a red triangle) was mounted on a post at a height of approximately 3 m to enhance LoRa communication range. It consisted of a Particle Boron Board powered by a rechargeable battery and a solar panel. Since the receiver listens to the channel continuously (24/7), battery-only operation would last only a few days. The solar panel helps extend its lifetime by charging the battery during daytime, allowing for uninterrupted operation even through the night. Before the deployment, we tested the communication range of all 12 modules and confirmed that they could successfully transmit to the receiver at distances up to 1 km. Each module’s microcontroller was programmed with a unique ID, enabling the receiver to demultiplex received packets accordingly. All modules transmitted packets using LoRa radios operating in the unlicensed 915 MHz spectrum. The radios were configured with a spreading factor of 10, bandwidth of 250 kHz, and a coding rate of 4/5. These parameters strike a balance between range, reliability, and data rate.

The deployment was left operational in the field for nearly one month, corresponding to approximately 30 days or 720 h of field operation. Most modules remained operational throughout the entire deployment period. During the first week, three modules temporarily stopped functioning due to rain-induced water leakage and abnormal current draw, which caused early battery depletion. After replacing the affected batteries and resecuring the modules, these nodes resumed operation and continued collecting data until the end of the deployment. The remaining modules operated continuously throughout the month-long study. The following subsections detail the sensing results collected during this period.

### 3.1. Temperature Results

**Figure 7:**
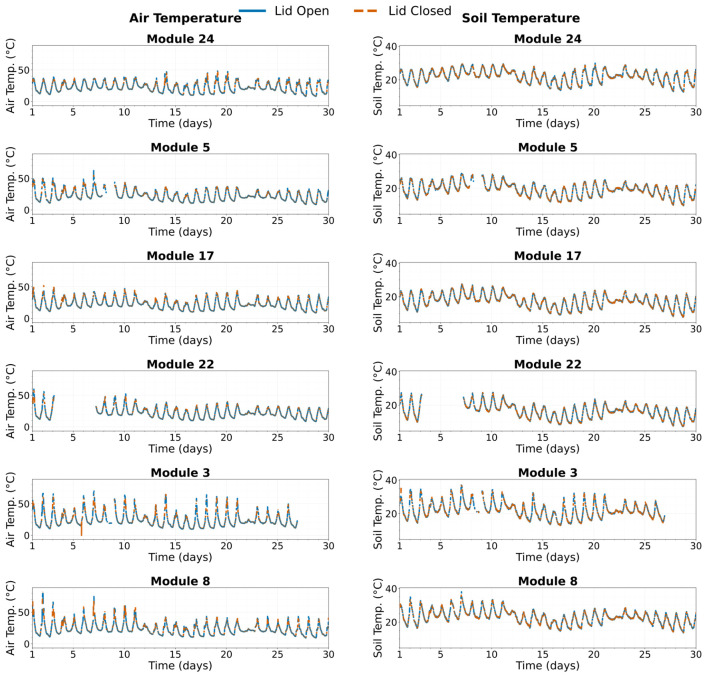
Temporal variations in air and soil temperatures measured by different modules distributed across field, capturing spatial variations.

**Figure 8:**
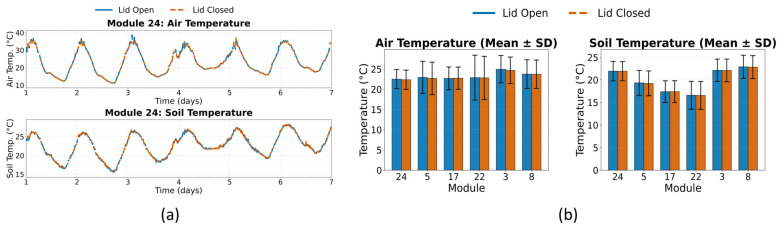
(**a**) One-week zoomed view from Module 24 showing air and soil temperature dynamics. (**b**) Mean ± SD summary across all modules.

Figure 7 shows the variations in air and soil temperature over time for different modules deployed across the field. Since the modules are spatially distributed, they capture both temporal and spatial variability in temperature. In the time-series plots, the solid blue segments correspond to periods when the chamber lid was open, while the dashed orange segments indicate the lid was closed. Figure 8a shows a zoomed one-week view for Module 24, where a clear and repeating diurnal pattern is observed. The temperature increases during the daytime and decreases during the night, repeating consistently across consecutive days. The soil temperature follows the same general daily trend as the air temperature, but with a much smaller fluctuation range, indicating the damping effect of soil thermal inertia.

A preliminary observation from the multi-module temperature measurements reveals that the soil temperature exhibits a significantly narrower range of fluctuations than air temperature. Among the six representative modules shown, Module 8 records the highest air temperature swing, ranging from 10 °C to 62 °C, corresponding to a 52 °C range, whereas Module 24 exhibits the smallest range at 29 °C. In contrast, soil temperature variations remain under 15 °C, with no module exceeding 35 °C. This discrepancy arises due to the thermal properties of air versus soil and the soil heat flux that creates lagged and damped temperature fluctuations in the soil compared to that in the air.

Figure 8b presents the mean ± 1 standard deviation (SD) summary of air and soil temperature across the deployed modules. The lid-open and lid-closed values are very close for both air and soil temperature, suggesting that the chamber lid state has minimal impact on the measured temperature. This indicates that the chamber design does not introduce a strong thermal bias between open and closed conditions. The relatively large SD, especially for air temperature, is expected because the measurements include repeated day–night cycles and weather-driven variations over the monitoring period.

In summary, across all modules, we observe a distinct diurnal sinusoidal pattern with a 24 h period: temperature peaks between midday and the afternoon when solar radiation is strongest, and drops at night, reaching a trough around midnight. The status of the lid, open or closed, appears to have minimal impact on both air and soil temperature, further supporting that the chamber allows effective thermal exchange regardless of lid position. Interestingly, around Day 16, a notable dip in air temperature is observed across all modules, correlating with rainfall and overcast conditions between Days 14–19. This validates the sensor system’s responsiveness to environmental drivers of soil gas fluxes. For some modules, such as Modules 5 and 22, discontinuities in the plots correspond to brief outages where the module went offline and later resumed operation. These month-long temperature observations demonstrate the system’s robustness, spatial resolution, and long-term reliability in capturing fine-grained temperature dynamics relevant to soil health monitoring.

### 3.2. Humidity and Pressure Results

**Figure 9:**
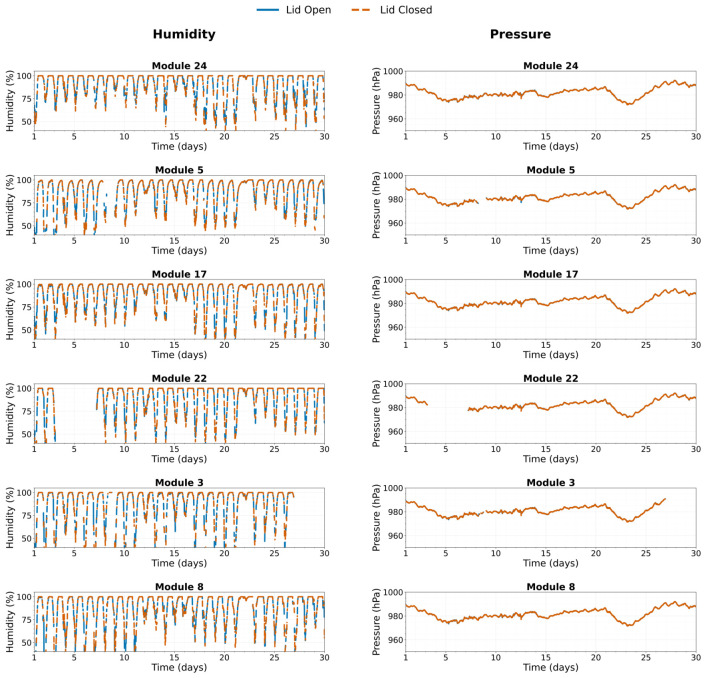
Temporal variations in relative humidity and air pressure measured by modules distributed across the field, capturing spatial and temporal variations during the deployment.

**Figure 10:**
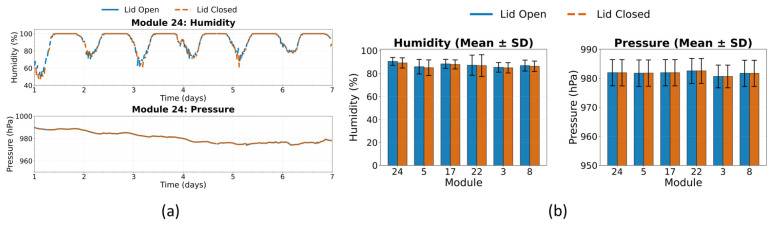
(**a**) One-week zoomed view of Module 24 showing relative humidity and air pressure dynamics. (**b**) Mean ± SD summary across all representative modules.

Figure 9 shows the relative humidity and air pressure measurements recorded by the modules distributed across the field over the full monitoring period. In the plots, the solid blue segments correspond to periods when the chamber lid was open, while the dashed orange segments indicate periods when the lid was closed. Figure 10a presents a zoomed one-week view for Module 24, while Figure 10b summarizes the mean ± SD values across all representative modules.

All modules show a similar trend in relative humidity. In the zoomed view shown in Figure 10a, humidity repeatedly increases toward 100% during the nighttime and decreases during the daytime. This pattern is mainly due to the diurnal temperature cycle: nighttime cooling increases relative humidity and promotes dew formation, while daytime solar radiation increases air temperature and reduces relative humidity. Soil water evaporation and plant activity also contribute to the local moisture dynamics. Across the full monitoring period shown in Figure 9, the minimum humidity varies across modules, with Modules 5 and 8 exhibiting values below 50% around Day 2.

Figure 10b shows that the mean humidity values during the lid-open and lid-closed periods are similar across the modules, indicating that lid status has only a minor effect on the overall humidity measurements. However, the relatively large SD reflects the strong natural day–night humidity cycle and weather-driven variations over the month-long deployment. Therefore, the observed spread in humidity is primarily attributable to environmental variability rather than inconsistent sensor behavior.

Air pressure, on the other hand, shows a highly consistent trend across all modules. Compared with humidity and temperature, pressure exhibits the strongest agreement among the spatially distributed modules, as expected for an atmospheric variable that changes relatively uniformly over the field scale. The pressure variation remains within a narrow range of approximately 20 hPa over the monitoring period. The mean ± SD summary in Figure 10b further confirms that the lid-open and lid-closed pressure values are nearly identical, indicating that lid status does not affect the pressure measurements. Overall, the humidity and pressure results demonstrate that the system captures both local microclimatic variations in humidity and spatially consistent atmospheric pressure trends across the deployment.

### 3.3. CO_2_ Emission Flux Rate Results

#### 3.3.1. Calibration Performance

As discussed in Section 2.5, we performed linear regression between the NDIR sensor readings and the benchmark measurements to calibrate each sensor module. The calibration models yielded an average coefficient of determination ($R^{2}$) of 0.996, with individual module values ranging from 0.994 to 0.998, indicating a strong fit. The average root mean squared error (RMSE) across all modules was 29.53 ppm. When normalized to each module’s CO_2_ concentration range, the RMSE percentage error ranged from 1.41% to 2.06%, further demonstrating the accuracy of the calibration.

#### 3.3.2. Variations in CO_2_ Flux Rates

**Figure 11:**
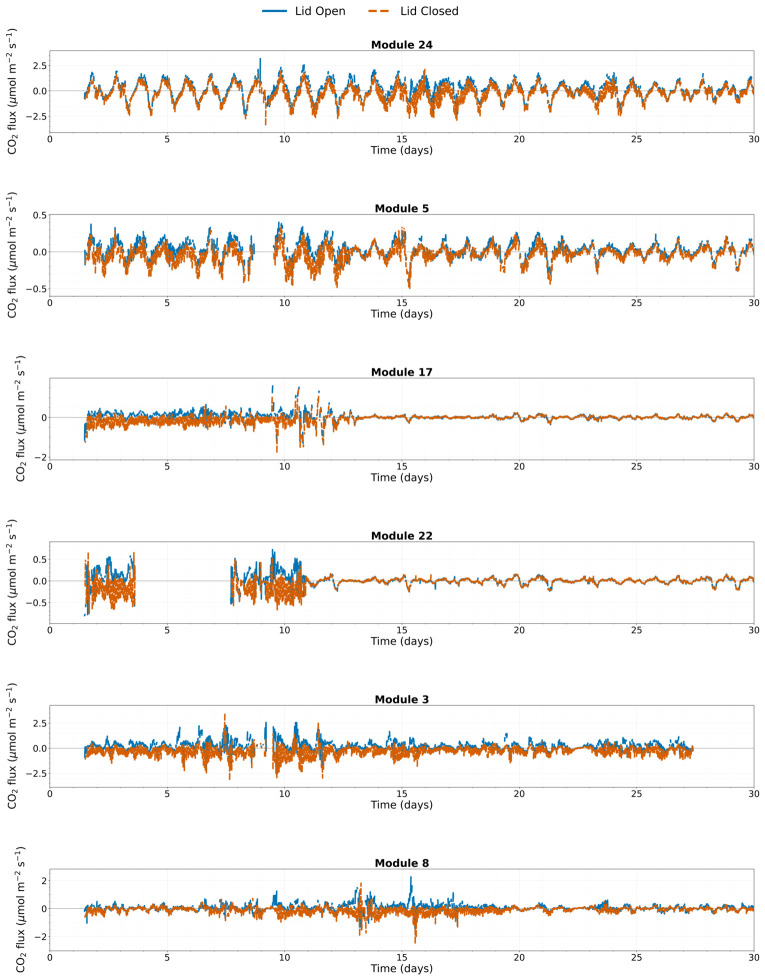
Temporal variations in CO_2_ emission flux measured by different modules distributed across the field, capturing spatial variations.

After calibration, we used the regression equation in Equation (1) to obtain corrected CO_2_ readings. These corrected readings were then used in Equation (2) to compute the CO_2_ emission flux rates. The flux rate trends for six representative modules are shown in Figure 11. The plotted traces use a moving-average smoothing window to improve visual clarity while preserving the temporal flux trends. Solid blue traces correspond to lid-open periods, while dashed orange traces correspond to lid-closed periods. As evident from Figure 11, the CO_2_ emission flux rate varies significantly across different modules. Module 24 has a range of approximately 4 μmol m^−2^ s^−1^, Modules 8 and 3 have a range of approximately 2 μmol m^−2^ s^−1^, Modules 5 and 17 have a range of approximately 1 μmol m^−2^ s^−1^, and Module 22 has a smaller range of approximately 0.4 μmol m^−2^ s^−1^. This shows that even within a relatively small field area, CO_2_ emission flux can vary substantially across different locations. This spatial heterogeneity underscores the importance of high-resolution flux monitoring even within small-scale agricultural plots.

**Figure 12:**
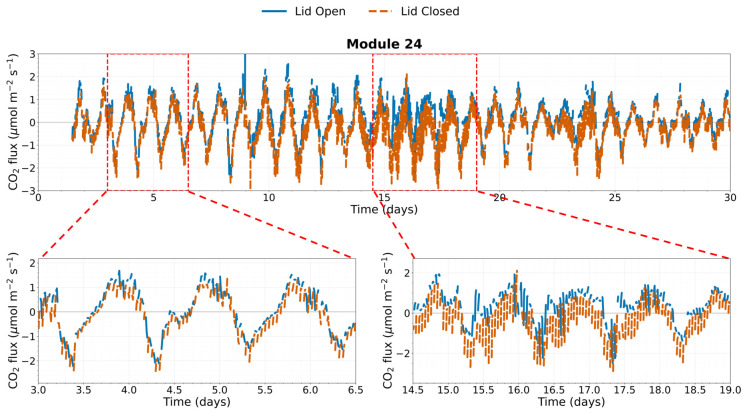
Zoomed CO_2_ emission flux for Module 24. The selected windows show distinct lid-dependent flux responses, with the open–closed mean flux difference increasing from 0.22 μmol m^−2^ s^−1^ during Days 3–6 to 0.65 μmol m^−2^ s^−1^ during Days 14–19.

Figure 12 zooms into the CO_2_ flux rate data for Module 24 around two representative time windows. These snapshots reveal two distinct yet related patterns in the temporal dynamics of CO_2_ emission. The first is a global diurnal trend, where the flux varies with an approximately daily periodicity. This behavior is consistent with environmental and biological drivers such as plant photosynthesis, plant and microbial respiration, and temperature-dependent soil activity. The second is a localized chamber-cycle response that aligns with the lid status of the chamber. When the chamber lid changes state, the headspace transitions between an accumulation phase and a ventilation or re-equilibration phase, producing a repeatable lid-dependent flux response. The open–closed mean flux differences in the highlighted windows were computed by averaging the smoothed flux values separately during lid-open and lid-closed periods within each selected time window, and then subtracting the lid-closed mean from the lid-open mean. The increase in this separation from 0.22 to 0.65 μmol m^−2^ s^−1^ indicates that the chamber-state response is not only detectable but also varies over time with changing environmental conditions.

**Figure 13:**
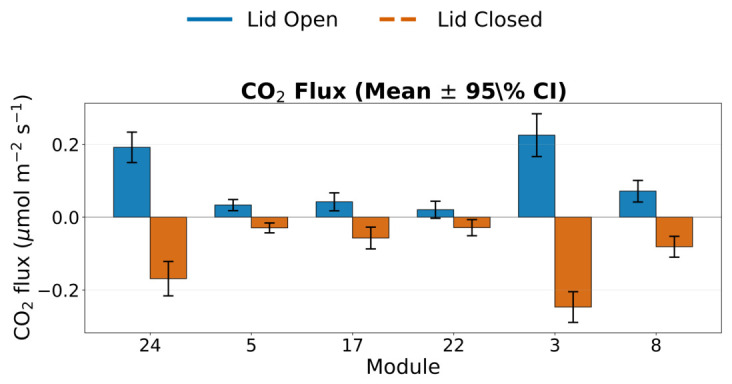
Mean ± 95% confidence interval of ${\mathrm{CO}}_{2}$ emission flux across deployed modules, showing distinct lid-dependent responses and spatial variability in flux magnitude.

Figure 13 summarizes the flux response across modules using mean ± 95% confidence intervals computed from daily averaged smoothed flux values. This statistical summary shows that the lid-open and lid-closed periods produce clearly separated responses across modules, while the magnitude of this separation varies spatially. Modules 24 and 3 show the largest flux magnitudes, whereas Modules 5, 17, 22, and 8 exhibit smaller responses. The confidence intervals indicate that these differences are repeatable across the monitoring period rather than being caused by isolated transient events.

The opposite signs observed between lid-open and lid-closed periods arise from the chamber measurement cycle and the sign convention used in the flux-processing pipeline. During one phase, CO_2_ accumulates in the chamber headspace, while during the other phase, the chamber is ventilated and the headspace returns toward ambient conditions. Therefore, the sign indicates the direction of the calculated concentration change according to Equation (2), rather than implying that the soil alternates between being a CO_2_ source and sink during every lid cycle. The important inference is that the system captures a repeatable and distinguishable chamber-state-dependent CO_2_ response, while the module-to-module differences reflect spatial variability in soil CO_2_ dynamics.

Similar trends were observed across other modules, though with varying amplitudes depending on local soil moisture, temperature, and microbial activity. These results highlight that, unlike existing flux measurement techniques that are either too sparse, such as chamber systems with manual sampling, or prohibitively expensive, such as eddy covariance towers, our solution offers a low-cost, scalable, and automated alternative. It captures flux rates at high spatial resolution across many modules and with fine temporal granularity, enabling detailed analysis of soil health and CO_2_ dynamics across diverse field conditions.

## 4. Discussions and Limitations

This work leveraged state-of-the-art, low-power, long-range communication protocols along with innovative sensor module design to monitor gas emission at a high spatial and temporal resolution. While the results demonstrate the promise of an inexpensive monitoring system that can scale to a large field deployment, there are few steps remain toward making it an autonomous monitoring system.

### 4.1. Power Consumption

Energy and battery life are bottlenecks in any outdoor settings. This is particularly true in environments with extreme weather conditions. In our current design, the sensor, microcontroller, and communication module were designed to minimize power consumption and in turn increase battery life. Further improvements could be made by optimizing the circuit board to reduce leakage current and unnecessary wake times, and by improving the power delivery for the motors. As part of our future work, we plan to improve the energy efficiency of each sensor module, and make them more resilient to extreme changes in weather conditions.

### 4.2. Wireless Communication

We used off-the-shelf LoRa transceivers and standard configurations in our deployment. Further experiments on various wireless configurations to maximize the number of messages sent, extend battery life, and improve communication range under varying environmental conditions would render this an autonomous system. We plan to explore long-term deployments to see the impact of environmental conditions of different sensors and the communication modules.

### 4.3. Further Analysis on Noise and Environmental Conditions

This feasibility study shows the promise of LoRa along with a low-cost CO_2_ chamber for wireless monitoring of soil respiration. However, further analysis is needed on the impact of the environmental conditions on the wireless performance. For instance, we observed missing data from several modules following a rain event during the first week of deployment. Water intrusion and loose connections temporarily interrupted their operation. The affected batteries were replaced, the connections were reattached, and the modules were resealed, after which they resumed operation. A more robust, leak-proof enclosure could reduce such interruptions in future deployments.

### 4.4. Calibration and Validation Limitations

We calibrated the CO_2_ sensor in this experiment, assuming that the temperature and pressure sensors had negligible measurement errors. To fully evaluate the accuracy of the soil CO_2_ flux in the field, it is necessary to compare our sensing system with other widely used methods, such as automated gas chamber systems that measure a similar footprint of the soil (e.g., LI-COR, Gasmet). In addition, the current soil moisture sensors used in the system are based on capacitance and used as an indicator for the relative moisture content of the soil. To make full use of the soil moisture and other ancillary environmental variables measured by our sensing module, further work to develop a calibration protocol for these sensors is needed.

### 4.5. Post-Deployment CO_2_ Calibration

The CO_2_ sensors were calibrated before field deployment, as described in Section 2.5. Post-deployment recalibration was not performed for this deployment; therefore, long-term sensor drift after field exposure could not be directly quantified from this dataset. However, the high pre-deployment calibration accuracy and the consistency of the environmental trends observed across modules support the feasibility of the sensing approach. Evaluating post-deployment recalibration and sensor drift over longer deployments remains an important direction for future work.

### 4.6. Limitations

While the proposed framework offers a low-cost alternative to large-scale measurement of carbon flux, the quality of data could deteriorate over time. It could require more frequent calibrations due to sensor malfunctioning. Further analysis on its applicability in other soil types, fields, and environmental conditions is needed to ensure a robust sensing system.

## 5. Conclusions

In this work, we developed and implemented a low-cost, battery-operated CO_2_ sensing system to obtain high-resolution data in the spatial and temporal domain. We carefully designed PCB layouts to improve the battery life of our sensing system in order to deploy in outdoor settings with minimal manual intervention. We incorporated an ultra-low-power microcontroller along with a long-range communication protocol, LoRa, to attain high spatial resolutions over long distances without significant manual labor. Our innovative PVC-based gas chamber design to house the sensing electronics allows for gas exchange. This design makes the chamber inexpensive and durable. Despite using low-cost sensors and simpler gas chambers, the RMSE across all modules was below 2.5%, showcasing the promise of this feasibility study. The field deployment demonstrated that the system can capture meaningful spatial and temporal variations in environmental parameters and CO_2_ emission flux. Temperature and humidity showed clear diurnal trends, while pressure remained consistent across modules. The lid-open and lid-closed summaries showed minimal differences for temperature, humidity, and pressure, indicating that the chamber design does not introduce significant bias in these measurements. In contrast, CO_2_ flux showed distinct lid-dependent responses and spatial variability: for example, Module 24 exhibited an open–closed mean flux difference increasing from 0.22 to 0.65 μmol m^−2^ s^−1^ between selected time windows. These findings demonstrate that the proposed system can resolve both short-term chamber dynamics and module-to-module spatial variability in soil CO_2_ emissions. In summary, this sensing system offers a low-cost alternative to gas chambers for CO_2_ monitoring; the lower cost will allow more researchers to reach a higher spatial resolution, further improving our understanding of gas emissions in a wide area. We deployed 12 sensor modules across eight stations in an agricultural field over multiple weeks and observed variations in temperature, pressure, humidity, and CO_2_ flux rates with fine spatial and temporal resolution.

## 6. Patents

The system described in this manuscript is related to U.S. Patent No. 12,529,686 B2, “Soil gas-flux measurement system,” issued 20 January 2026, and assigned to the Wisconsin Alumni Research Foundation.

## Figures and Tables

**Table 1:** Calibration coefficients and performance metrics for the ${\mathrm{CO}}_{2}$ sensor modules used in the field analysis. The intercept $\beta_{0}$ and slope $\beta_{1}$ correspond to the linear calibration model $y=\beta_{0}+\beta_{1}x_{1}$, where $x_{1}$ is the raw sensor reading and *y* is the benchmark ${\mathrm{CO}}_{2}$ concentration.

Module ID	$R^{2}$	RMSE (ppm)	$\beta_{0}$ (ppm)	$\beta_{1}$	Error (%)
24	0.998	21.84	11.98	0.97	1.43
5	0.994	37.45	−59.28	0.90	2.06
17	0.995	27.47	90.30	0.97	1.87
22	0.994	37.55	58.84	1.06	2.06
3	0.997	20.75	73.13	1.04	1.41
8	0.995	32.11	51.52	1.01	1.92
**Mean **	**0.996**	**29.53**	**37.75**	**0.99**	**1.79**

## Data Availability

The code and PCB design files supporting this study are publicly available in the UW-CONNECT Soil-Sensors GitHub repository at https://github.com/UW-CONNECT/Soil-Sensors- (accessed on 16 July 2026). Additional data supporting the findings of this study are available from the corresponding author upon reasonable request.
